# Microbiota during pregnancy and early life: role in maternal−neonatal outcomes based on human evidence

**DOI:** 10.1080/19490976.2024.2392009

**Published:** 2024-08-19

**Authors:** Alessio Fasano, Benoit Chassaing, Dirk Haller, Eduard Flores Ventura, Maria Carmen-Collado, Nitida Pastor, Omry Koren, Roberto Berni Canani

**Affiliations:** a Mucosal Immunology and Biology Research Center, Mass General Brigham, Harvard Medical School, Harvard T.H. Chan School of Public Health, Boston, MA, USA; b European Biomedical Research Institute of Salerno (EBRIS), Salerno, Italy; c Microbiome-Host Interactions, Institut Pasteur, INSERM, Université Paris Cité, Paris, France; d Mucosal Microbiota in Chronic Inflammatory Diseases, INSERM, CNRS, Université de Paris, Paris, France; e Nutrition and Immunology, School of Life Sciences, Technical University of Munich, Freising, Germany; f Department of Biotechnology, Institute of Agrochemistry and Food Technology – Spanish National Research Council (IATA-CSIC), Valencia, Spain; g Department of Medical Affairs, Clinical Research, Mead Johnson Nutrition, Evansville, IN, USA; h Azrieli Faculty of Medicine, Bar-Ilan University, Safed, Israel; i Department of Translational Medical Science, and ImmunoNutritionLab at Ceinge Advanced Biotechnologies Research Center, and European Laboratory for Investigation of Food Induced Diseases, University of Naples Federico II, Naples, Italy

**Keywords:** Gut microbiome, epigenetics, short-chain fatty acids, precision medicine, probiotics, prebiotics, postbiotics, live biotherapeutics

## Abstract

Here, we explored the vast potential of microbiome-based interventions in preventing and managing non-communicable diseases including obesity, diabetes, allergies, celiac disease, inflammatory bowel diseases, malnutrition, and cardiovascular diseases across different life stages. We discuss the intricate relationship between microbiome and non-communicable diseases, emphasizing on the “window of opportunity” for microbe–host interactions during the first years after birth. Specific biotics and also live biotherapeutics including fecal microbiota transplantation emerge as pivotal tools for precision medicine, acknowledging the “one size doesn’t’ fit all” aspect. Challenges in implementation underscore the need for advanced technologies, scientific transparency, and public engagement. Future perspectives advocate for understanding maternal−neonatal microbiome, exploring the maternal exposome and delving into human milk's role in the establishment and restoration of the infant microbiome and its influence over health and disease. An integrated scientific approach, employing multi-omics and accounting for inter-individual variance in microbiome composition and function appears central to unleash the full potential of early-life microbiome interventions in revolutionizing healthcare.

## Introduction

The incidence of health deterioration associated with non-communicable diseases (NCDs) has been on an upward trajectory. Data projects that by 2050, several NCDs, such as diabetes and ischemic heart disease, will be among the leading causes of diseases.^1^ The primary health system’s preventative measures involve minimizing exposure to risk factors. However, addressing these risk factors poses both economic and technical challenges for public and private health systems.^2^ Reducing exposure to risk factors and adopting healthy dietary habits, such as increasing the intake of whole grains, fermented foods, and soluble fibers while reducing the consumption of refined grains and processed meat, will modulate the gut microbiota and reduce the risk of NCDs.^3,4^

The importance of microbiome modulation for health and disease management shows promise in preventing and managing or treating NCDs.^5,6^ These alterations are causally implicated in physiological, immunological, and metabolic processes and ultimately in various inflammatory diseases, including autoimmune diseases.^7–9^ Acting as a mediator, the gut microbiome vertical transmission and the sharing of environments with NCD-related microbial reservoirs may influence the onset and progression of diseases. This suggests that it transforms NCDs from noncommunicable to communicable,^10–12^ and the breakdown of the symbiotic relationship between the intestinal microbiome and its host emerges as a potential cause for NCDs. Additionally, the gut microbiome can influence brain functions through neuroinflammation processes driven by inappropriate antigen trafficking, which can include nutrients.^10,13–15^

Research on the human microbiome, encompassing intestinal and extra-intestinal microbiotas, appears vital for understanding the causes and complications of NCDs.^14^ There is indeed a shared understanding that exerting better control over the composition and function of an individual’s microbial ecosystem can be advantageous for promoting the production of metabolites derived from microbes.^16^ Here we aim to give an overview of the potential use of pre-, pro-, and post-biotics and dietary interventions in disease attenuation and management throughout different life stages, emphasizing the relevance of the ‘window of opportunity’ during early infancy and showcasing the microbiome’s potential to revolutionize healthcare and enhance well-being during pregnancy, lactation, and long-lasting effects in later stages in life.

## Prenatal factors and early-life maternal-fetal microbiome

### Impact of prenatal physiological changes on maternal microbiome and pregnancy outcomes

The hormonal, immunological, and metabolic changes during pregnancy exert an influence on microbiome and clinical outcomes with potential implications for maternal and infant well-being.^17^ Physiological changes during pregnancy are related to shifts in maternal microbial diversity,^18,19^ and this has been related to adverse pregnancy outcomes and obstetric diseases such as gestational diabetes mellitus (GDM).^20,21^ These outcomes suggest a relationship between microbiota and maternal physiological changes that may infer complication pre-, peri-, and post-partum in both the mother and offspring^22^ (Table 1). As an example, a metagenomic sequencing analysis of 749 women from the InSPIRe cohort in France showed that maintaining lower microbial diversity and lower vaginal *Lactobacillus crispatus* abundance during the last trimester of pregnancy may be linked to preterm delivery, and the overall diversity of vaginal microbiota could be used as an indicator of preterm delivery risk.^23^ Another recently published study demonstrated that GDM, which is currently diagnosed toward the end of the second trimester, can be predicted from as early as week 12 using clinical and immunological data and microbiome characteristics.^20^ Additionally, changes in maternal oral microbiome have also been linked with adverse outcomes including preterm pre-labor rupture of fetal membranes, preterm birth, and low birth weight, hypertension, and chorioamnionitis.^24^ Furthermore, maternal periodontitis may also influence the risk of asthma in the offspring.^25^ Nevertheless, some positive effects have been observed in the presence of microbial aromatic hydrocarbons and extracellular vesicles in cord blood and amniotic fluid for conditions such as diabetes, food allergy, neonatal necrotizing enterocolitis (NEC), or autism spectrum disorder.^26^

**Table 1.** Microbiota changes during pregnancy and maternal and fetal clinical conditions: human-based evidence.

Microbiota derived changes during pregnancy	Clinical conditions	Reference
Vaginal microbial lower diversity and decreased abundance of *Lactobacillus crispatus*.	Preterm delivery.	23
Maternal oral microbiota dysbiosis with prevalent pathogenic microbes, leading to maternal periodontitis and gingivitis.	Pre-labor rupture of membranes, preterm birth, and low birth weight, hypertension and chorioamnionitis, and risk of asthma in the offspring.	24,25
Presence of microbial aromatic hydrocarbons and extracellular vesicles in cord blood and amniotic fluid.	Increased fetal growth, amelioration of diabetes, food allergy, neonatal necrotizing enterocolitis, or autism spectrum disorder	26
Increased proinflammatory cytokines, significant differences in UniFrac dissimilarity, Shannon diversity, and the presence of *Fusobacteria* and *Deferribacteres*.	Gestational diabetes mellitus	20
Increased intestinal *Proteobacteria* and *Actinobacteria* with decreased abundance of *Prevotella*, *Varibaculum*, *Lactobacillus*, and *Porphyromonas*.	Preeclampsia	27,28
Increased intestinal *Bacteroides*, *Faecalibacterium*, and *Lachnospira* with decreased *Enterococcus* and *Acinetobacter*.	Fetal growth restriction	29

### Exploring the bidirectional relationship between maternal microbiota and pregnancy complications

The interplay between host inflammatory processes and microorganisms are involved in conditions such as GDM, pre-eclampsia, and preterm birth.^17^ During the first trimester, an increase in inflammatory markers, a decrease in microbial metabolites, such as short chain fatty acids (SCFA), and microbial shifts have been observed.^30^ These changes in inflammatory and microbial biomarkers may provide an early opportunity to predict and prevent pregnancy complications. In addition, the women microbiome may not only be related to the women's health but also to the fetal health and development as well as pregnancy outcomes. Despite the debate on in utero microbiome exposure,^31^ maternal microbial metabolites and also microbial extracellular vesicles have been shown to affect the fetoplacental unit. For example, several groups have recently demonstrated the effect of maternal microbiota and microbial metabolites on placental development in mice,^32,33^ and Li et al. reported an *in-utero* metabolome originating from the maternal microbiome in humans.^17,34^ In addition to deepening our understanding of this research area, we also need to gain more knowledge of how well defined pre/pro/post-biotics given to the mother can improve fetal development and shape offspring health.

## Perinatal factors and neonatal microbiome

### Influences of maternal factors on neonatal gut colonization and health outcomes

The assembly of the infant’s gut colonization is a pivotal process, influencing their health and development,^35^ especially during the first 2 years post-partum when the microbial composition tends to mature into an adult-like microbiota.^36^ *Bifidobacterium* and *Bacteroides* emerge as critical factors in the early establishment of the infant gut, exerting a substantial impact on health outcomes.^37^ Additionally, bioactive components in Human Milk (HM) function in a symbiotic manner, encompassing both microbiota and prebiotics, such as HM oligosaccharides (HMOs).^38^ Furthermore, the constituents of HM play a regulatory role in infant growth,^39^ as well as in shaping the composition of the intestinal microbiome,^40^ and fortifying the immune system.^41^ Yet, maternal factors, such as diet, antibiotic usage, genetics, and the mode of delivery exert a notable influence on the composition of HM and its microbial diversity.^42^ These fluctuations, in consequence, possess the potential to influence infant outcomes, underscoring the rationale for maternal interventions to address neonatal health proactively.

### Methodological challenges and future directions in studying human milk composition and its impact on infant health

Several methodological challenges have surfaced in the examination of HM composition and its repercussions on infant health, microbiome engraftment, and development. These include the precise and reliable quantification of both known and novel components through untargeted omics approaches.^43^ Additional challenges in studying HM involve daily and feeding variations, hindmilk and foremilk distinctions, diurnal fluctuations, ethnicities, and compositional heterogeneity among other covariates.^44^ Future initiatives should aim at maternal-neonatal microbiota care, vertical transmission of antibiotic resistance genes, microbial modulation, and HM-infant microbiota restoration. The importance of live biotherapeutics, probiotics, genome-wide association studies, and regulatory factors cannot be overstated. Identifying microbial markers and leveraging beneficial compounds from HM and feces offer promising pathways for improving mother-infant well-being. Table 2 shows examples of potential biotherapeutics derived from milk components and supplements that could be used to fortify formula milk that have shown potential benefits for infants. Knowledge of humans in this field remains scarce, and studies have mostly been conducted in animals, although with positive effects shown,^32^ clinical studies in humans are yet to be conducted.

**Table 2.** Examples of milk sourced compounds and supplements with potential functional properties for infant health and development.

Milk sourced compounds and supplements with potential functional properties	Potential benefit	Reference
Fat globules and Lactoferrin	Neurodevelopment, growth, and reduced risk of respiratory adverse events and diarrhea.	45,46
Betaine	*Akkermansia* abundance modulation and long-term metabolic health.	47
Butyrate	Food allergy	48
Glycosaminoglycans	Infection modulation and intestinal metabolism.	49
*Lacticaseibacillus rhamnosus* GG	Metabolic dysfunction, allergic manifestations, and atopic dermatitis in children.	50–52
Microbial strains of *Bifidobacterium, Lactobacillus, Bacteroides, Enterococcus, Streptococcus.*	Reduce the risk of allergic asthma development.	53
HMO	Necrotizing Enterocolitis	54
Immunoglobulin A	Patterns of bacterial recognition and immune training contribute to passive immunity in infants by attaching to the mucosal surface of enterocytes and neutralizing potential threats directly.	55,56

## Microbiota dynamics post-birth: health evidence

### Early-life microbiota dynamics and the impact on long-term health outcomes

Literature suggests that the first year after birth constitutes a critical time frame that may impact long-term health outcomes,^57^ in which various mechanisms have been described linking modulation of gut microbiota, gut permeability, epigenetics, and immune responses. The establishment of early microbiota plays a pivotal role in determining susceptibility to dysbiosis, wherein the absence of specific microbial species in infants may elevate the likelihood of various diseases. Notably, maintaining a diverse and beneficial microbiota is imperative, where its loss has been shown to increase predisposition to chronic inflammatory conditions, such as allergies, inflammatory bowel diseases (IBD), autoimmune diseases, metabolic disorders, neurodegenerative and neurodevelopmental disorders, and cancer.^58^ Environmental factors encompass the exposome, understood as the exposures that an individual comes across throughout life, such as dietary habits and food additives found in ultra-processed foods (UPFs), including, select synthetic dietary emulsifiers that could exert profound influences on the intestinal microbiota in a way that potentiate chronic intestinal inflammation and associated downstream diseases.^59–61^ These can induce shifts in microbial balance, either compositionally or functionally, thereby shaping the trajectory of disease development.^62,63^ Of note, these recent studies on diet–microbiota interaction highlighted the central role played by microbiota encroachment within the normally sterile inner mucus layer, opening innovative therapeutic approaches for the prevention of microbiota-driven chronic inflammatory conditions.^63^

### Interventions and future directions for improving post-birth microbiota health

Moreover, it is crucial to underscore that within the first 2 years after birth, HM emerges as one of the primary nutrient sources, along with weaning and the introduction to solid foods. These factors are critical for the subsequent development of gut microbiota and the reduced risk of the onset of food allergies.^64^ Yet, in instances where HM is unavailable, the importance of fortified formula milk enriched with probiotics and prebiotics could be a valid alternative, where numerous studies have highlighted their critical contribution to delivering essential nutrients and cultivating a favorable microbial environment.^65^ More research is needed to understand early microbial colonization, environmental factors, and subsequent health outcomes, in order to advance our understanding of their applications to improve clinical outcomes during this critical developmental window.^66^ Future investigations will have to consider confounding factors like antibiotic use, probiotics, and dietary information. Global initiatives such as the Human Microbiome Action^67^ and the MicrobiomeSupport Association^68^ aim to standardize methodologies for studying the microbiome’s impact on health, emphasizing the ongoing need for refinement and standardized approaches to analyze and interpret microbiome composition and function. Moreover, interdisciplinary approaches involving multi-omics data, artificial intelligence, and big data analysis are in development. Additionally, precision nutrition, incorporating genetics, epigenetics, microbiome, and health data holds promise for addressing health disparities,^60,69^ but necessitates further exploration for effective applications. Shifting focus from individual bacterial strains to the functionality of the microbiome is crucial for targeted interventions. There are incompletely understood aspects, such as the immune memory and its interplay with dysbiosis in NCDs. Furthermore, the role of postbiotics, defined as “a preparation of inanimate microorganisms and/or their components that confers a health benefit on the host”, as stated by the International Scientific Association for Probiotics and Prebiotics (ISAPP),^70^ represent a promising avenue for study and potential health interventions, where conditions like autism and depression present opportunities, with inflammation as a focal point for potential prevention and treatment, contributing to a more nuanced understanding of microbiome-related therapeutics.

## Microbiome-NCD connections unraveled

### Microbiota-driven pathophysiological mechanisms and disease susceptibility

The pathophysiological connections still remain unveiled with regards to the microbiota, gut, and brain and conditions such as allergies, IBD, obesity, depression, autism, and celiac disease.^71^ An underlying mechanism involved could be mediated by the interaction between microbiota-derived metabolites with Toll-like receptors and NOD-like receptors, playing a role in antimicrobial peptide production, immune cell recruitment, and the onset of pathologies such as NEC.^72^ This suggests that personalization accounting for microbiota composition is relevant to prevent and treat disease. Furthermore, the loss of specific microbial species in infants may increase the risk of diseases such as celiac disease,^73^ emphasizing the significance of preserving a diverse microbiota. Additionally, the pathological mechanisms that denote disease in early life can also be explained by increased gut permeability and zonulin-mediated mechanisms^74^ that can precede the onset of the disease,^75^ where an already existing epithelial barrier leak, and increased zonulin production may facilitate susceptibility to translocation of microbiota-produced lipopolysaccharides (LPS).^76^ Research shows that to improve our understanding of these mechanisms, we need to delve into microbiome−host immune system interactions depicting luminal ligands such as T cell receptors and antigens, microtubule-associated proteins, and pattern recognition receptors, bile acids and farnesoid receptors, SCFA, and formyl peptide receptors.^77^

### Western dietary habits and their impact on gut microbiota and non-communicable diseases

Western dietary habits characterized for being high in fats, sugars, calories, and highly processed foods or UPFs have been linked to changes in gut microbiota and NCDs.^78^ UPFs are understood as those with five or more ingredients along with salt, oil, sugar, fats, other substances such as hydrolyzed protein, modified starches, thickeners, and various additives whose purpose is to enhance sensory stimulus.^79^ These can impact disease through modulation of the intestinal microbiota by inferring dysbiosis and facilitating the production of metabolites and other factors that can activate neuronal, endocrine, and immune pathways. In addition, theories such as “germ-organ theory of NCDs”^16^ outline the effect of dysbiosis as one of the causes of NCDs due to the changes in bacteria, and consequently, changes in the production and concentration of microbiota-derived metabolites. Preclinical data from animal studies have indicated that food additives such as carrageenan, carboxymethyl cellulose (CMC), and Polysorbate 80 (P80) can facilitate the entry of bacteria into the mucosal layer, modulate intestinal microbiota diversity, and consequently increase the risk of disease.^80^ We still do not understand the long-term effects, and some individuals may be more susceptible than others to the detrimental effects of dietary emulsifiers, necessitating a personalized approach. In pediatric populations exposed to UPF, those exposed to high sugar and color additives have been seen to have detrimental long-term effects on health, increasing risk to hyperactivity, impairments in hippocampal-dependent episodic memory, and obesity.^81^ Research seems to be gapping knowledge on the impact of UPF components on health in relation to microbiota in the long term at different stages in life, and the challenge radiates in exploring this phenomenon accounting for the inter-individual microbial compositional variance.^82^ Nevertheless, research conducted on animals has reported the potential use of probiotic strains of *Akkermansia muciniphila*, such as the #BAA-835 (ATCC) strain, in mitigating the effects of dietary emulsifiers on the host and its microbiota.^63^ This probiotic strain may contribute to an increased immune response and mucus secretion, thereby facilitating mucus turnover and leading to a reduction in risk.^63^ Research in this line could drive product reformulation, tailored dietary guidelines, and probiotic recommendations.

## Microbes in NCDs: attenuation and management

### Strategies for attenuating pathogenic effects of gut microbiota in non-communicable diseases

An adequate gut microbial ecosystem throughout all stages in life could reduce the risk of NCDs onset, where metabolite production has been linked to inflammatory and metabolic diseases.^83^ Bacteria can act opportunistically and as pathobionts, expressing pathogenic traits under specific conditions, making infants predisposed to diseases.^84^ Beneficial gut bacteria play a crucial role in preventing opportunistic infections through various means, such as antimicrobial synthesis, SCFA production, bile acid metabolism modulation, promotion of mucin formation, and maintaining immune balance in the mucosal lining.^85^ To this end, via multiple mechanisms that inhibit pathobionts, increase commensal bacteria, and modify bacterial metabolome, probiotics. synthetic communities, phages, diet, and fecal microbiota transplantation (FMT) administration may reduce the pathogenic effect of opportunistic, pathobiont bacteria, and ultimately persistent dysbiosis and increased gut barrier permeability.^86^ Clinical trials that use FMT in adults show that the transition phase in dietary interventions understood as the step between change from baseline and stabilization represent a window of opportunity for effective probiotic mediated weight-loss dietary interventions, and management of recurrent *Clostridioides difficile* infection,^87^ where those that do not use FMTs lead to higher participant rebounds of adiposity, insulin resistance, and metabolic syndrome after intervention when compared to dietary interventions that do consider FMTs.^88,89^ In this line, Food and Drug Administration (FDA) approvals of FMT therapies for recurrent *C. difficile* infections include the RBX2660 (live biotherapeutic),^90^ and the Ser 109 (synthetic biotherapeutic).^91^ Furthermore, the use of vaginal microbiota transplants after antibiotics treatment to remove harmful pathogens successfully achieve donor strain engraftment and address severe vaginal dysbiosis.^92^

### Personalized microbiome-driven approaches for managing non-communicable diseases

Microbiome signatures are presently adequate for characterizing population-based risk,^93^ suggesting their potential sufficiency in stratifying individual disease risk. Consequently, in microbiome research, it is crucial to consider personalization and intervention across various levels, from individual to subgroup levels. This involves a thoughtful selection of case/control groups to ensure meaningful research outcomes and informed decision-making while accounting for the inter-individual variance of microbial traits.^94^ Furthermore, it is essential to highlight that microbial diversity fluctuates throughout life, with circadian oscillations,^95^ emphasizing the need for precise time collection of samples for accurate results.

## Diet and microbiome: health implications

### Impact of maternal diet on infant microbiome and immune development

Maternal diet significantly influences infant outcomes, impacting both the newborn’s microbiome and the immune system. However, existing research has not fully considered microbial inter-individual variance in establishing optimal maternal nutrient recommendations during pregnancy.^96^ Maternal diet exerts a profound impact on both mothers and infants, yielding negative effects such maternal undernutrition contributing to stunting by 2 years of age and increased risk of atherogenic lipid profile.^97,98^ Emerging research underscores that the quality of the diet is linked to distinctive gut microbiota profiles,^99^ as well as HM bioactive compounds that associate with different dietary components. Nevertheless, covariates such as the mode of delivery and antibiotic intake are also factors involved in shaping microbial composition.^42^

### Interventions and strategies for modulating microbiota composition through maternal dietary change

Additionally, during pregnancy and lactation, short-term maternal dietary changes have been shown to influence the infant’s gut microbial functional pathways.^100^ Studies indicate that manipulating the microbiota can be effectively achieved through interventions such as via HM or specifically formulated substitutes, dietary fiber, and probiotic supplements (Table 2). When personalized, these interventions not only impact the composition of your microbes but also influence the functionality of the microbiota, leading to changes in metabolite production and, ultimately, impacting human health.^101^ Moreover, focusing on interventions in early life, particularly during the ‘window of opportunity,’ holds special significance. Data indicates that the use of prebiotics, probiotic strains, synbiotics, and postbiotics in formula milk have the potential to influence the establishment and development of infant gut microbiota.^102,103^ Comprehending the dynamics of the mother-milk-infant triad is vital for tailoring interventions aimed at the long-term health of both the mother and offspring. Current challenges include replicating the maturation of immunity and the microbiota seen in infants who are breastfed and delivered vaginally – these infants serve as the benchmark for comparison.

### Microbial interventions for attenuation and management: challenges and opportunities

Challenges related to studying HM composition, including its complexity as a biofluid, as well as compositional variations within a day, between feeds, and among hindmilk and foremilk, neonate gender dependency, and the dynamic nature of HM during lactation, pose significant hurdles, highlighting the need for advanced technologies.^104^ Disease prediction based on microbiome data encounters challenges, with individual variations often masking disease specificity, thus preclude making therapy predictions.^105^ These complexities contribute to the challenges associated with implementing microbial interventions for disease prevention. Moreover, the uncertainty surrounding the recommendations in general for the use of pre- or probiotics for attenuating or managing infant diseases, due to the lack of conclusive evidence and the current non-consensus recommendation by scientific societies, further adds to the challenges in implementing microbial targeted interventions. Nevertheless, the potential utilization of -biotics by the population requires scientific transparency, effective communication, public engagement, and trust-building.^106^ This is essential to ensure the acceptance and understanding of the relationship between humans and microbes and the potential benefits they exert on human health. Challenges persist in achieving consistent effects in trials and to satisfy regulatory requirements, mainly due to the use of different technologies and to the barriers to consider a microbial strain as probiotic, where these must survive the digestive tract, compete against other microbes and antimicrobial activity, and once the bacteria has survived all the aforementioned barriers, they have to engraft and compete against the already existing core of microbes within the host´s microbiota.^107^ Research has reiteratively shown heterogeneous use of methodologies,^108^ thus precluding the possibility to jointly shape population-based recommendations, where the targets, clinical conditions, ingredient, durations, dosage, and matrix of preparations have been different.

## Future perspectives and challenges

By considering future perspectives and challenges related to exploring microbiota-driven approaches for NCD prevention and treatment, it becomes imperative to focus on safeguarding the maternal-neonatal microbiota during pregnancy and investigating the maternal exposome. To this end, we need randomized control trials that account for interindividual variations and microbiota-based stratification not only in early life, but there is also a lack of research in the field of aging. Additionally, understanding the short- and long-term health outcomes in infants resulting from early exposures is crucial, where the effect of UPFs and its ingredients including food additives and their effect on microbiota and derived clinical outcomes remain an area that requires further investigation. Furthermore, delving into HM, infant formula, complementary feeding, genetic predisposition, and the restoration of infant microbiota emerges as a promising avenue. A comprehensive approach is vital for effectively addressing pediatric allergies, given their potential shared mechanisms with conditions like depression, IBD, and autism. The epigenetic mechanisms, particularly those involving the production of immunomodulatory components, such as interleukin-4, stand out as focal points in ongoing allergy research. As we move forward, an integrated scientific approach, such as those employing multi-omics analysis through artificial intelligence approaches applied to prospective birth cohorts, will be pivotal in unraveling the complexities of these future considerations and meeting the associated challenges head-on.

## Conclusion

The intricate relationship between microbiome and NCDs highlights the potential of microbiome-based interventions across all life stages. From prenatal influences on the fetal microbiome to perinatal factors shaping the infant’s microbial landscape and the critical first 1,000 days post-partum, the pivotal role of microbial dynamics in health and development becomes evident. It is clear that the interventions with probiotics, prebiotics, synbiotics, postbiotics, and most importantly, proper nutrition in modulating the microbiome for improved health outcomes, represent a path toward precision medicine. The “one size doesn’t fit all” aspect is crucial, acknowledging the diverse influences on individual microbiomes and the need for personalized approaches. The challenges in implementation, highlighted in the study, underscore the necessity for advanced technologies, scientific transparency, effective communication, and public engagement. Future perspectives call for a comprehensive understanding of maternal-neonatal microbiota, investigation of the maternal exposome, and exploration of HM’s role in restoring infant microbiota. An integrated scientific approach, incorporating multi-omics and addressing challenges head-on, will be essential for realizing the full potential of microbiome-based interventions in revolutionizing healthcare and enhancing well-being. In essence, early-life microbiome interventions hold substantial potential for precision medicine and primary prevention strategies to ameliorate the burden of NCDs.

## Data Availability

Data sharing is not applicable to this article as no new data were created or analyzed in this study.
